# Flipping the Script: Measuring Both Performance Validity and Cognitive Ability with the Forced Choice Recognition Trial of the RCFT

**DOI:** 10.1177/00315125211019704

**Published:** 2021-05-22

**Authors:** Kaitlyn Abeare, Kristoffer Romero, Laura Cutler, Christina D. Sirianni, Laszlo A. Erdodi

**Affiliations:** 1 Department of Psychology, University of Windsor, Windsor, Ontario, Canada; 2 School of Social Work, University of Windsor, Windsor, Ontario, Canada

**Keywords:** Rey Complex Figure Test, forced choice recognition, performance validity, embedded validity indicators

## Abstract

In this study we attempted to replicate the classification accuracy of the newly introduced Forced Choice Recognition trial (FCR) of the Rey Complex Figure Test (RCFT) in a clinical sample. We administered the RCFT*_FCR_* and the earlier Yes/No Recognition trial from the RCFT to 52 clinically referred patients as part of a comprehensive neuropsychological test battery and incentivized a separate control group of 83 university students to perform well on these measures. We then computed the classification accuracies of both measures against criterion performance validity tests (PVTs) and compared results between the two samples. At previously published validity cutoffs (≤16 & ≤17), the RCFT*_FCR_* remained specific (.84–1.00) to psychometrically defined non-credible responding. Simultaneously, the RCFT*_FCR_* was more sensitive to examinees’ natural variability in visual-perceptual and verbal memory skills than the Yes/No Recognition trial. Even after being reduced to a seven-point scale (18-24) by the validity cutoffs, both RCFT recognition scores continued to provide clinically useful information on visual memory. This is the first study to validate the RCFT*_FCR_* as a PVT in a clinical sample. Our data also support its use for measuring cognitive ability. Replication studies with more diverse samples and different criterion measures are still needed before large-scale clinical application of this scale.

## Introduction

The validity of clinical decisions based on neuropsychological test scores hinges on the assumption that examinees gave maximal (or at least typical) cognitive effort during testing (Bigler, 2015; Dandachi-FitzGerald et al., 2016; Merten & Merckelbach, 2013; Roor et al., 2016). Historically, clinicians assumed valid performance by default, and verified it only through behavioral observations (e.g., through observed level of cooperation, apparent ability and willingness to follow instructions). However, the limitations of clinical judgment for detecting non-credible responding have long been demonstrated empirically (Dandachi-FitzGerald et al., 2017; Heaton et al., 1978), and this realization has led to the development and use of objective measures for differentiating valid from invalid performances. Early performance validity tests (PVTs) were free-standing instruments, often based on the forced choice recognition (FCR) paradigm. With the advent of managed care, assessors faced pressure to abbreviate test batteries without compromising test data quality. As expansive, multi-trial free-standing PVTs became harder to justify to third party medical insurers, they were gradually replaced by embedded validity indicators (EVIs) that served the PVT function from within traditional neuropsychological ability tests.

Although EVIs have tended to exhibit inferior signal detection relative to traditional PVTs (Lau et al., 2017) and they have sometimes been criticized for conflating ability and effort (Bigler, 2012; Leighton et al., 2014), their advantages have included (a) cost-effectiveness; (b) reduced mental stamina demands for young or medically/emotionally fragile examinees (Lichtenstein et al., 2017); and (d) an inconspicuousness that made them more difficult for examinees to detect (An et al., 2019; Berger et al., 2019) and, therefore, more resistant to coaching (Brennan et al., 2009; Erdal, 2004; Lippa, 2018; Weinborn et al., 2012). Of equal importance, EVIs protect assessors from the appearance of a confirmation bias when PVT use seems to have been motivated by clinician expectations of examinee malingering (Boone, 2013).

In recent decades EVI research has proliferated. A popular method of EVI development has been to add a FCR trial to existing memory tests, such as the California Verbal Learning Test (Delis et al., 2000), California Verbal Learning Test – Children’s Version (Lichtenstein et al., 2017; 2018); Rey Auditory Verbal Learning Test (Poreh et al., 2016), and Hopkins Verbal Learning Test (Abeare, Hurtubise et al., 2020; Cutler et al., 2021). Following this trend, Rai et al. (2019) introduced an FCR trial to the Rey Complex Figure Test (RCFT); this RCFT*_FCR_* was placed 10 minutes after the end of the standard administration protocol.

The original RCFT (Rey, 1941), in conjunction with its add-on trials and scores (Meyers & Meyers, 1995; Lu et al., 2003; Reedy et al., 2013), offers clinicians a range of tools for evaluating the visual-perceptual and memory skills of adults and children. The paper and pencil copying task (i.e., the reproduction of the complex figure while the stimulus remains exposed to the examinee) can detect impairments in visuospatial construction, fine-motor coordination, and planning/organization skills. Clinically significant variations in performance may signal deficits in encoding, storage, and retrieval processes (Shin et al., 2006).

Meyers and Meyers (1995) first introduced a Yes/No Recognition trial using 24 single line drawings consisting of 12 targets and 12 foils. For this task, examinees are instructed to circle the shapes that they recognize as being part of the original figure. The examinee’s raw score is the number of correct decisions made [i.e., the sum of true positives (targets circled) and true negatives (foils not circled)], ranging from 0 to 24. The Yes/No Recognition trial proved useful as an EVI (Shura et al., 2016; Whiteside et al., 2011). Lu et al. (2003) developed an equation that provided a single-number estimate of the validity of the response set based on multiple RCFT scores, and this equation was subsequently cross-validated (Blaskewitz et al., 2009; Reedy et al., 2013; Shura et al., 2016).

For the newer scale, the RCFT*_FCR_,* the initial validation study was*_,_* based on the experimental malingering paradigm (Rai et al., 2019). The RCFT*_FCR_* had comparable classification accuracy to the Yes/No Recognition trial, despite its different administration format. Instead of the Yes/No Recognition format of presenting examinees with a number of shapes scattered on a page, the RCFT*_FCR_* consists of 24 items, each containing a target and a foil. Within each pair, the examinee is asked to *identify the one that was part of the original figure*. As such, the score ranges from 0 (foil chosen every time) to 24 (target chosen every time). (Complimentary digital copies of the Rey Complex Figure and the RCFT*_FCR_* trial are available to qualified readers through the senior author.)

The RCFT*_FCR_* was specifically developed to appear difficult to the examinee. Unlike most FCR trials that present the examinee with an easy choice between a familiar target and a fairly obvious intrusion error as the alternative option, the RCFT*_FCR_* includes a number of items that are genuinely challenging, for two reasons. First, given the growing awareness that a test based on FCR is likely to be a PVT – or a trap to avoid – the authors made the RCFT*_FCR_* harder to identify as an EVI and thus, harder for sophisticated malingerers to evade. Second, the authors expressed hope that, following a non-traditional EVI presentation, this FCR might double as an actual measure of cognitive abilities (i.e., perceptual skills and visual memory). To achieve these goals, they engineered the stimulus properties (i.e., discriminability) of the target and foil to increase the cognitive demands of the task (i.e., both items seem comparably plausible at first glance). In the original study with no clinical sample, the only circumstantial evidence supporting this feature was a finding of equivalent mean scores from healthy participants when comparing the Yes/No Recognition and RCFT_*FCR*_ trials.

As a novel EVI, the RCFT*_FCR_* has had limited empirical support. To date, it has only been examined among cognitively intact students within an experimental malingering paradigm, and this has limited the generalizability of the findings to clinical populations (Giromini et al., 2019; Lindstrom et al., 2011; Sullivan & King, 2010; Viglione et al., 2019). In fact, Rai et al. (2019) emphasized the importance of testing their newly introduced instrument in a clinical sample, and they ended their paper with a call for replication among patients with confirmed or suspected genuine memory deficits. The present study, designed to examine the classification accuracy of the RCFT*_FCR_* in patients clinically referred for neuropsychological assessment, is an answer to that call.

Given criticisms that it is easier to differentiate healthy controls from those instructed to feign deficits (experimental malingering paradigm) than credible patients with genuine cognitive deficits from patients with co-occurring genuine deficits and invalid performance (Fuermaier et al., 2017; Giromini et al., 2018; Merten & Rogers, 2017; Stevens et al., 2008; van Helvoort et al., 2019), we predicted an attenuation in the RCFT*_FCR_*’s classification accuracy when the RCFT_*FCR*_ was applied to a clinical population. However, based on the results of the original study, we hypothesized that the Yes/No Recognition and the RCFT_*FCR*_ trials would be comparably sensitive to fluctuations in cognitive ability. Finally, we included a sample of students who were incentivized to perform well in order to address a separate limitation of the experimental malingering paradigm – variable motivation of research volunteers to demonstrate their maximal ability level as a control group (An et al., 2012; Hurtubise et al., 2020; Lace et al., 2020; Roye et al., 2019). Given this added incentivizing, we expected the student controls in our sample to outperform Rai et al.’s (2019) control group.

## Method

### Participants

Our clinical sample consisted of a consecutive case sequence of 52 patients referred for neuropsychological assessment to the last author’s private practice in order to evaluate their cognitive and emotional functioning in the context of determining eligibility for disability benefits. As such, these participants can be considered as positive for having external incentives to appear impaired (Slick et al., 1999). The main inclusion criteria for these participants were the administration of the added RCFT_*FCR*_ trial and their informed consent for their clinical data to be used for research purposes. Participants’ mean age was 37.9 years (*SD* = 13.0; range: 18–63). Their mean education level was 11.1 years (*SD* = 2.0; range: 6–14). Most (88.5%) were Caucasian (5.8% Black, 3.8% mixed, and 1.9% Aboriginal), right-handed (82.7%), and male (57.7%).

Our student sample consisted of 83 undergraduate students enrolled in a third-year course on psychometrics. As part of their grade, they were required to demonstrate credible performance during in-class assignments. This contingency was instituted to ensure that students took their assignments seriously, maximizing the pedagogical value of these experiential learning opportunities. Therefore, they had external incentives to perform well. Inclusion criteria were a valid administration of the RCFT*_FCR_* trial, and at least one of the following free-standing PVTs: the first trial of the Test of Memory Malingering (TOMM-1; Tombaugh, 1996) or the Word Choice Test (WCT; Pearson, 2009). Since the RCFT Yes/No Recognition Test and RCFT_*FCR*_ (*n* = 83), the TOMM-1 (*n* = 67) and the WCT (*n* = 75) were administered on different days, the sample size for these tests differed (as denoted), reflecting the natural fluctuation in student attendance. As noted below in Procedures, students also gave informed consent for their performance data to be used in this research and had the opportunity to opt out from research participation (i.e., withdraw their consent for their anonymized test scores being used for academic research). Only de-identified data were used for this study. The university’s Research Ethics Board approved the secondary use of the test scores for research purposes.

### Measures

In addition to the RCFT, all patients completed a core battery of neuropsychological tests, including the Matrix Reasoning, Vocabulary, Digit Span and Coding subtests of the Wechsler Adult Intelligence Scale – Fourth Edition (WAIS-IV; Wechsler, 2008), the Trail Making Test (TMT A & B; Reitan, 1955); the Hopkins Verbal Learning Test – Revised (HVLT-R; Brandt & Benedict, 2001), letter, category and emotion word fluency (Abeare et al., 2017; Gladsjo et al., 1999), Stroop test of the Delis-Kaplan Executive Function System (D-KEFS, 2001), Grooved Pegboard Test (GPB; Lafayette Instrument, 2015), Complex Ideational Material (CIM) of the Boston Diagnostic Aphasia Battery (Goodglass et al., 2001); Conners’ Continuous Performance Test – Third Edition (CPT-3; Conners, 2015), the Rey Fifteen-Item (Rey-15) and Word Recognition Test (WRT; Rey, 1941); Boston Naming Test – Short Form (Erdodi et al., 2017), the Clock Drawing Test (CDT; Rouleau et al., 1992), the Patient Health Questionnaire (PHQ-9; Spitzer et al., 1999); the Generalized Anxiety Disorder Seven (GAD-7; Spitzer et al., 2006), the Five-Variable Psychiatric Screener (V-5; Erdodi, Jongsma, et al., 2020; Sirianni et al., 2021) and the Behavior Rating Inventory of Executive Function (BRIEF; Roth et al., 2005). Demographically adjusted T-scores for the TMT, CIM, GPB and verbal fluency were calculated using the norms by Heaton et al. (2004). The main free-standing PVTs were the TOMM-1 and the WCT enhanced with the time-to-completion (T2C) cutoff.

#### Validity Composites (EI-5s)

To complement the free-standing PVTs and to monitor the modality specificity effect (Lace et al., 2020; Rai & Erdodi, 2019; Schroeder et al., 2019), we developed two validity composites by aggregating individual EVIs, using the methodology developed by Erdodi (2019). The first one was based on tests that appeared to measure memory (EI-5*_MEM_*), representing the modality-congruent criterion; the other was based on tests of processing speed (EI-5*_PSP_*), representing the modality in-congruent criterion. The presence of an engineered method variance in criterion PVTs allowed for a more rigorous test of the classification accuracy of both RCFT recognition trials by minimizing the risk of spurious findings and improving ecological validity.

First, we recoded each of the five constituent PVTs onto a four-point ordinal scale such that a score that passed the most liberal cutoff was coded as zero, a score that failed the most conservative cutoff was coded as three, failing the next most liberal cutoff was coded as one, and failing the next most conservative cutoff was coded as two (see Table 1). We computed the value of the EI-5s by summing the recoded constituents, yielding a range from 0 (patient passed all five components at the most liberal cutoff) to 15 (patient failed all five components at the most conservative cutoff). An EI-5 value ≤1 was considered an overall *Pass*, as it signaled, at most, one marginal failure. EI-5 values 2 and 3 were difficult to interpret, as they might have represented either a couple of marginal failures or a single failure at a conservative cutoff. Neither of these combinations provided sufficient evidence to deem the entire profile invalid; therefore, this range was labeled Borderline and was excluded from analyses requiring a dichotomous outcome. However, an EI-5 ≥4 indicated either multiple failures at the liberal cutoff, or at least two at the conservative cutoff, crossing the line into the non-credible range (Pearson, 2009).

**Table 1.** Components of the EI-5s and Base Rates of Failure at Given Cutoffs (Clinical Sample).

	EI-5 Values					EI-5 Values			
EI-5*_MEM_*	**0**	**1**	**2**	**3**	EI-5*_PSP_*	**0**	**1**	**2**	**3**
Components	Pass	Fail	FAIL	FAIL	Components	Pass	Fail	FAIL	FAIL
CIM*_BDAE_*	>9	8–9	7	≤6	Animals	>31	26–31	24–25	≤23
*Base rate*	*71.7*	*18.9*	*3.9*	*5.7*	*Base rate*	*73.6*	*15.3*	*5.7*	*5.7*
									
DS*_WAIS_*	>6	5–6	3–4	≤2	CD*_WAIS_*	>5	5	4	≤3
*Base rate*	*68.6*	*18.9*	*5.9*	*3.9*	*Base rate*	*64.2*	*13.2*	*13.2*	*9.4*
									
FCR*_HVLT-R_*	12	11	9–10	≤8	TMT-A	>33	23–33	20–22	≤19
*Base rate*	*83.0*	*5.7*	*9.4*	*1.9*	*Base rate*	*67.9*	*20.8*	*7.5*	*3.8*
									
Rey-15 FR	>11	7–11	5–6	≤4	VAR*_CPT-3_*	<65	*65*–*74*	75–79	≥80
*Base rate*	*75.5*	*13.2*	*5.7*	*5.7*	*Base rate*	*71.7*	*13.2*	*9.4*	*5.7*
									
Rey WRT	>6	5–6	4	≤3	Word*_D-KEFS_*	>5	4–5	2–3	1
*Base Rate*	*71.7*	*17.0*	*5.7*	*5.7*	*Base rate*	*69.8*	*9.5*	*9.5*	*11.3*

*Note*. Shading represents the change in confidence in correctly classifying a given score as *invalid* (darker means more likely to be invalid); EI-5*_MEM:_* Erdodi Index Five - Memory; EI-5*_PSP:_* Erdodi Index Five – Processing Speed; CIM*_BDAE:_* Complex Ideational Material subtest of the Boston Diagnostic Aphasia Battery raw score (An et al., 2019; Erdodi, 2019; Erdodi et al., 2016; Erdodi & Roth, 2017); DS*_WAIS:_* Digit Span subtest of the Wechsler Adult Intelligence Scale (age-corrected scaled score; Erdodi & Abeare, 2020; Erdodi & Lichtenstein, 2017; Hurtubise et al., 2020; Reese et al., 2012; Shura et al., 2019; Spencer et al., 2013; Webber & Soble, 2018; Whitney et al., 2009); FCR*_HVLT-R_*: Forced choice recognition trial of the Hopkins Verbal Learning Test – Revised (Abeare, Hurtubise, et al., 2020; Cutler et al., 2021); Rey-15 FR: Rey Fifteen-Item Test free recall (Boone et al., 2002; Lezak, 1995; Merten et al., 2005; O’Bryant et al., 2003; Poynter et al., 2019; Russeler et al., 2008); Rey WRT: Rey Word Recognition Test (Bell-Sprinkel et al., 2013; Goworowski et al., 2020; Love et al., 2014; Nitch et al., 2006; Smith et al., 2014); Animals: Animal fluency T-score using norms by Heaton et al., 2004 (Hurtubise et al., 2020; Sugarman & Axelrod, 2015); CD*_WAIS_*: Coding subtest of the Wechsler Adult Intelligence Scale (age-corrected scaled score; Erdodi & Abeare, 2020; Erdodi, Abeare, et al., 2017; Erdodi & Lichtenstein, 2017; Etherton et al., 2006; Inman & Berry, 2002; Kim et al., 2010; Trueblood, 1994); TMT-A: Trail Making Test – Part A T-score using norms by Heaton et al., 2004 (Abeare, Sabelli, et al., 2019; Ashendorf et al., 2017; Erdodi & Lichtenstein, 2020); VAR*_CPT-3:_* Variability score of the Conners’ Continuous Performance Test – Third Edition (Erdodi, Pelletier, et al., 2018; Erdodi, Roth, et al., 2014; Ord et al., 2020); Word*_D-KEFS_*: Word Reading trial of the Delis-Kaplan Executive Function System (Arentsen et al., 2013; Boskovic et al., 2018; Donders & Hayden, 2020; Egeland & Langfjaern, 2007; Eglit et al., 2019; Erdodi, Sagar, et al., 2018; Guise et al., 2014).

The majority of the participant samples (55-60%) scored in the Passing range on both versions of the EI-5. A quarter of the patients (26-28%) scored in the Failing range. Consistent with previous research (An et al., 2019; Erdodi & Abeare, 2020; Erdodi, Green, et al., 2019; Erdodi & Rai, 2017; Erdodi, Taylor, et al., 2019; Rai & Erdodi, 2019), the EI-5s were significant predictors of the two free-standing PVTs. An EI-5*_MEM_* score ≥4 was specific to failing the TOMM-1 or the WCT (.90–.96), at .65 sensitivity. The EI-5*_PSP_* produced a similar combination of sensitivity (.69–.70) and specificity (.86–.96).

#### Visual-Perceptual Ability Composite (VPA-3)

The VPA-3 was designed to serve as the ability measure counterpart to the EI-5s. Thus, the VPA-3 was conceived as a composite of visual-perceptual ability, created as a criterion measure for evaluating the RCFT_*FCR*_’s sensitivity to the examinees’ natural fluctuations in perceptual skills. As its name suggests, the VPA-3 consisted of three tests designed to assess perceptual reasoning, visual scanning or visuomotor speed: the Matrix Reasoning and Coding subtests of WAIS-IV and TMT-A. Similar to the EI-5s, these components of the VPA-3 were recoded onto a five-point scale in which zero corresponded to two *SD*s below the mean (i.e., Impaired range), whereas four corresponded to two *SD*s above the normative mean (i.e., a Very Superior range). A score of two represented the Average range (see Table 2).

**Table 2.** Components of the Visual-Perceptual Ability Composite (VPA-3) in the Clinical Sample

		VPA-3 value				
Test	Scale	0	1	2	3	4
CD*_WAIS_*	ACSS	≤4	5–7	8–12	13–15	≥16
MR*_WAIS_*	ACSS	≤4	5–7	8–12	13–15	≥16
TMT-A	T	≤30	31–43	44–56	57–69	≥70

*Note*. CD*_WAIS_*: Coding subtest of the Wechsler Adult Intelligence Scale (age-corrected scaled score); MR*_WAIS_*: Matrix Reasoning subtest of the Wechsler Adult Intelligence Scale (age-corrected scaled score); TMT-A: Trail Making Test – Part A T-score using norms by Heaton et al. (2004).

The VPA-3 is analogous to the WAIS-IV index scores (Verbal Comprehension, Perceptual Reasoning, Working Memory and Processing Speed) as it combines information from multiple tests into a single-number summary of the broader construct. Multivariate measurement models based on aggregating different sources of data have been shown to be superior to single test scores (Abeare, Erdodi, et al., 2020; Pearson, 2009; Tyson et al., 2018). For the specific purpose of this study, the VPA-3 served as the criterion for calibrating the RCFT*_FCR_* as a measure of visual recognition memory (i.e., *ability* test).

### Procedure

Patients completed a clinical interview and a comprehensive neuropsychological test battery. Tests were administered and scored by trained psychometrists under the supervision of a licensed clinical neuropsychologist. Students were administered the RCFT with the FCR trial, the TOMM-1 and WCT as a group in a classroom. Failing the validity cutoff embedded within a given assignment resulted in a 0.2–0.6% penalty applied to the final grade, depending on the actual score. In addition, the instructor continuously emphasized the educational value of the in-class assignments to encourage full engagement. As noted earlier, students had the opportunity to opt out from research participation (i.e., withdraw their consent for their anonymized test scores being used for academic research). Only de-identified student data were used for the purposes of this research, and the protocols for testing both participant groups were approved by the Research Ethics Board of the university.

### Data Analysis

When relevant, we computed the base rate of failure (BR*_Fail_*; i.e., the percent of the sample that failed a given cutoff). The prevalence of the condition of interest (in this context, BR*_Fail_*) is a descriptive statistic that is important for understanding classification accuracy in general (Wald & Bestwick, 2014) and in the context of performance validity assessment specifically (Abeare, Messa, et al., 2019). Although area under the curve (AUC) is useful for comparing overall classification accuracy across models (Altman & Bland, 1994; Fawcett, 2006; Marzban, 2004), its clinical relevance has been called into question (Hand, 2009; Lobo et al., 2008; Wald & Bestwick, 2014). Therefore, sensitivity and specificity values were also computed around relevant cutoffs. In the context of performance validity assessment, specificity is the most important parameter that determines the clinically recommended cutoffs, whereas sensitivity is sacrificed in the interest of minimizing the false positive rate. The lowest acceptable specificity value is .84 (Larrabee, 2003), although values ≥.90 are desirable (Roberson et al., 2013). The main inferential statistic was the *t*-test, two-proportions *z*-test, Levene’s test of homogeneity of variance, AUCs with 95% CI, and Pearson’s product-moment correlations (*r*_xy_). All tests were two-tailed; alpha-level was set at .05. Effect size estimates were expressed in Cohen’s *d* and squared correlation coefficients (*r*_xy_^2^).

## Results

### Neuropsychological Functioning of the Clinical Sample

The patient sample’s performance on the Vocabulary (*M* = 8.1), Matrix Reasoning (*M* = 7.6) and Coding (*M* = 6.9) subtests of the WAIS-IV was in the Low Average range. The mean raw score on their RCFT Copy trial was 28.0. Performance on the acquisition (*M*_T-score_ = 32.6) and delayed free recall (*M*_T-score_ = 33.0) trials of the HVLT-R fell in the Borderline range. The mean raw score on the CDT was 8.5 out of 10. Performance on the TMT-A was in the Low Average range (*M*_T-score_ = 40.8). Dominant hand GPB performance, letter (FAS) and category (animal) fluency were in the Low Average range (*M*_T-score:_ 40.6–41.2). Self-reported depression on the PHQ-9 was in the Severe range (*M* = 16.2); self-reported anxiety on the GAD-7 was in the Moderate range (*M* = 13.7). The General Executive Composite on the BRIEF was in the clinical range (*M*_T-score_ = 73.8). Self-reported depression, anxiety and pain on the V-5 fell within the Moderate range (42.0–63.7).

### RCFT Yes/No Recognition Versus RCFT_*FCR*_ Scores for Clinical and Student Samples

#### Effects of Demographic Variables and Intra-Individual Differences

In the clinical sample, scores on the Yes/No Recognition and RCFT*_FCR_* trials were independent of examinees’ age (*p* = .434 and .728), education (*p* = .059 and .144) or sex (*p* = .237 and .977). Also, a repeated measures *t*-test revealed no significant difference between scores on the Yes/No Recognition trial (*M* = 19.3, *SD* = 2.2) and the RCFT*_FCR_* trial (*M* = 19.3, *SD* = 2.9): *t*(51) = 0.18, *p* = .859. These two recognition trials were positively correlated [*r*(52) = .37, *p* = .001]. One patient scored below chance level (<12) on the RCFT*_FCR_*.

In the student sample, the mean performance on the RCFT*_FCR_* trial (*M* = 22.1, *SD* = 1.8) was significantly higher than on the Yes/No Recognition trial (*M* = 21.3, *SD* = 1.6): *t*(82) = 3.90, *p* < .001, *d* = 0.43 (medium effect). As with the clinical sample, the two recognition trials were positively correlated [*r*(83) = .62, *p* < .001], but no participant scored below chance level on either RCFT recognition trial.

#### Sensitivity to PVT Failure

Interestingly, in the patient sample, there was no significant difference between patients who passed and those who failed the TOMM-1 on either of the RCFT recognition trials (Table 3). However, among patients, failing the WCT was associated with significantly lower performances on both RCFT recognition trials (*d*: 0.82–1.30, large effects). Similarly, patients who failed the EI-5s produced lower scores on both the Yes/No Recognition and the RCFT*_FCR_* trial. However, the effect was more pronounced on the EI-5*_MEM_* (*d*: 1.13–1.63, large) than on the EI-5*_PSP_* (*d*: 0.89–1.09, large). The effect size associated with failing the EI-5s was greater for the RCFT*_FCR_* (*d*: 1.09–1.63, large) than for the Yes/No Recognition trial (*d*: 0.89–1.13, large).

**Table 3.** Independent *t*-Tests Comparing Performance on the RCFT Yes/No and FCR Trials as a Function of Sample and Passing or Failing the Criterion PVTs.

	Criterion				RCFT Y/N recognition					RCFT *_FCR_*			
Sample	PVT		*n*	%	M	*SD*	*p*	*d*		M	*SD*	*p*	*d*
Clinical	TOMM-1	Pass	31	58.5	19.7	*2.1*	.087	–		19.6	*2.9*	.330	–
		Fail	22	41.5	18.7	*2.3*				18.8	*3.9*		
													
	WCT	Pass	29	54.7	20.1	*1.8*	.005	0.82		20.7	*1.8*	<.001	1.30
		Fail	24	45.3	18.4	*2.3*				17.4	*3.1* ^a^		
													
	EI-5*_MEM_*	Pass	32	69.6	19.9	*1.9*	<.001	1.13		20.2	*2.4*	<.001	1.63
		Fail	14	30.4	17.6	*2.0*				16.2	*2.5*		
													
	EI-5*_PSP_*	Pass	29	65.9	19.9	*1.7*	.005	0.89		20.4	*2.2*	.001	1.09
		Fail	15	34.1	17.9	*2.7* ^a^				17.2	*3.5* ^a^		
													
Student	TOMM-1	Pass	61	91.0	21.3	*1.6*	.413	–		22.4	*1.6*	.099	–
		Fail	6	9.0	20.7	*2.9* ^a^				21.2	*2.6* ^a^		
													
	WCT	Pass	53	70.7	21.4	*1.4*	.747	–		22.4	*1.7*	.085	–
		Fail	22	29.3	21.3	*1.7*				21.7	*1.4*		

*Note*. ^a^Levene’s test of homogeneity of variance *p* < .05; PVT: Performance Validity Test; RCFT: Rey Complex Figure Test; Y/N: Yes/No; FCR: Forced Choice Recognition raw score; TOMM-1: Trial 1 on the Test of Memory Malingering (Denning, 2012; Fazio et al., 2017; Greve et al., 2006, 2009; Jones, 2013; Kulas et al., 2014; Martin et al., 2020; Powell et al., 2004; Rai & Erdodi, 2019; Webber et al., 2018); WCT: Word Choice Test [Fail defined as accuracy score ≤47 (Barhon et al., 2015; Davis, 2014; Erdodi, Kirsch, et al., 2014; Pearson, 2009) or time-to-completion ≥156 seconds (Erdodi & Lichtenstein, 2021; Erdodi, Tyson, et al., 2017; Zuccato et al., 2018)]; EI-5*_MEM:_* Erdodi Index Five – Memory (*Fail* defined as ≥4); EI-5*_PSP:_* Erdodi Index Five – Processing Speed (*Fail* defined as ≥4).

### RCFT Variables as EVIs

#### Clinical Sample

The RCFT Copy trial was a significant predictor of all four criterion PVTs (Table 4). The first cutoff to reach the .90 specificity standard was ≤25.0, with .33–.43 sensitivity. At ≤23, specificity improved (.93–.97) at a reasonable cost to sensitivity (.29–.36). At ≤20, the Copy trial reached perfect specificity.

**Table 4.** Classification Accuracy of the EVIs Within the RCFT Against Criterion PVTs in the Clinical Sample.

				Criterion PVT							
				TOMM-1		WCT		EI-5 *_MEM_*		EI-5 *_PSP_*	
RCFT				SENS	SPEC	SENS	SPEC	SENS	SPEC	SENS	SPEC
Variable	Statistic	Cutoff	BR*_Fail_*	41.5		45.3		30.4		34.1	
Copy	AUC (95 CI)			.68 (.53–.83)		.72 (.58–.86)		.72 (.56–.88)		.68 (.51–.85)	
		≤25	26.9	.43	.84	.39	.83	.38	.81	.40	.82
		≤24	20.8	.41	.94	.33	.90	.38	.94	.40	.90
		≤23	15.1	.32	.97	.29	.97	.36	.97	.33	.93
		≤20	9.4	.23	1.00	.21	1.00	.21	1.00	.27	1.00
											
Y/N Rec	AUC (95 CI)			.64 (.48–.80)		.71 (.56–.85)		.81(.67–.94)		.73 (.54–.91)	
		≤18	30.2	.45	.81	.46	.83	.57	.78	.53	.83
		≤17	19.2	.27	.87	.33	.93	.43	.91	.47	.93
		≤16	17.0	.27	.90	.29	.93	.43	.94	.47	.97
		≤15	5.7	.09	.97	.13	1.00	.14	1.00	.20	1.00
FCR	AUC (95 CI)			.60 (.44–.76)		.83 (.71–.95)		.92 (.84–1.00)		.79 (.63–.96)	
		≤18	32.7	.52	.81	.70	.97	.92	.88	.73	.93
		≤17	21.2	.29	.84	.43	.97	.54	.91	.47	.93
		≤16	15.4	.19	.87	.35	1.00	.38	.94	.40	.96
		≤15	11.5	.14	.90	.26	1.00	.31	.94	.27	.96
Both Y/N Rec		≤18	19.2	.33	.90	.39	.97	.54	.94	.53	.96
& FCR		≤17	13.5	.19	.90	.26	.97	.31	.94	.33	.96
		≤16	7.7	.10	.94	.17	1.00	.15	.97	.27	1.00

*Note*. EVI: Embedded validity indicators; PVT: Performance Validity Test; RCFT: Rey Complex Figure Test; Y/N Rec: Yes/No recognition raw score; FCR: Forced Choice Recognition raw score; TOMM-1: Trial 1 on the Test of Memory Malingering (Denning, 2012; Fazio et al., 2017; Greve et al., 2006, 2009; Jones, 2013; Kulas et al., 2014; Martin et al., 2019; Powell et al., 2004; Rai & Erdodi, 2019; Webber et al., 2018); WCT: Word Choice Test [Fail defined as accuracy score ≤47 (Barhon et al., 2015; Davis, 2014; Erdodi, Kirsch, et al., 2014; Pearson, 2009) or time-to-completion ≥156 seconds (Erdodi & Lichtenstein, 2020; Erdodi, Tyson, et al., 2017; Zuccato et al., 2018)]; EI-5*_MEM:_* Erdodi Index Five – Memory (*Fail* defined as ≥4); EI-5*_PSP:_* Erdodi Index Five – Processing Speed (*Fail* defined as ≥4); BR*_Fail:_* Base rate of failure (% of the sample that failed a given cutoff); SENS: Sensitivity; SPEC: Specificity.

The RCFT Yes/No Recognition trial was a significant predictor of three criterion PVTs, narrowly missing the TOMM-1. The ≤18 cutoff failed to reach minimum specificity against any of the criterion PVTs. Lowering the cutoff to ≤17 notably improved specificity (.87–.93), at .27–.47 sensitivity. Making the cutoff even more conservative (≤16) produced marginal improvements in specificity (.90–.97) at negligible cost to sensitivity (.27–.47). Further lowering the cutoff (≤15) reached the point of diminishing return: small gains in specificity (.97–1.00) and a notable decline in sensitivity (.09–.20).

The RCFT*_FCR_* was a significant predictor of three of the criterion PVTs. The ≤18 cutoff achieved minimum specificity standards (.92–.97) against all criterion PVTs but the TOMM-1 (.81), at a wide range of sensitivity (.52–.92). Lowering the cutoff to ≤17 resulted in trivial gains in specificity (.84–.97) but a precipitous drop in sensitivity (.29–.54). Making the cutoff even more conservative (≤16) produced the predictable trade-off: further improvement in specificity (.87–1.00) and decline in sensitivity (.19–.40).

As a last step, the Yes/No Recognition trial and the RCFT*_FCR_* were combined. *Pass* on the combined measure was defined as a score above the cutoff on either of the two; *Fail* was defined as a score below the cutoff on both. The combined cutoff of ≤18 achieved the minimum specificity standard against all criterion PVTs (.90–.97), at .33–.54 sensitivity. Lowering the cutoff to ≤17 was the point of diminishing returns: no change in specificity, but a notable drop in sensitivity (.19–.33). Making the cutoff more conservative (≤16) consolidated specificity (.94–1.00) at a proportional cost to sensitivity (.10–.27).

Although there was a high rate of agreement (83-87%) between the Yes/No Recognition and the RCFT*_FCR_* trial at ≤16 and ≤17, 8% of the sample that passed the former failed the latter. Conversely, 6–10% of patients passed RCFT*_FCR_* but failed Yes/No Recognition at the same cutoff. As such, the two RCFT recognition trials were non-redundant EVIs.

#### Student Sample

Neither the Yes/No Recognition nor the RCFT*_FCR_* trial was a significant predictor of passing or failing the TOMM-1. The Yes/No Recognition trial also produced a non-significant AUC (.64, 95% CI: .39–.90) against the WCT. However, the RCFT*_FCR_* trial was a significant predictor of passing or failing the WCT (AUC = .68, 95% CI .55–.81). The only conceivable cutoff (≤18) was highly specific (.93–.97) but insensitive (.05–.33) on both trials, against both criterion PVTs. Failing the ≤18 cutoff on both the Yes/No Recognition and the RCFT*_FCR_* trials was associated with perfect specificity.

### The Effect of External Incentive vs. Genuine Impairment

**Figure 1.**
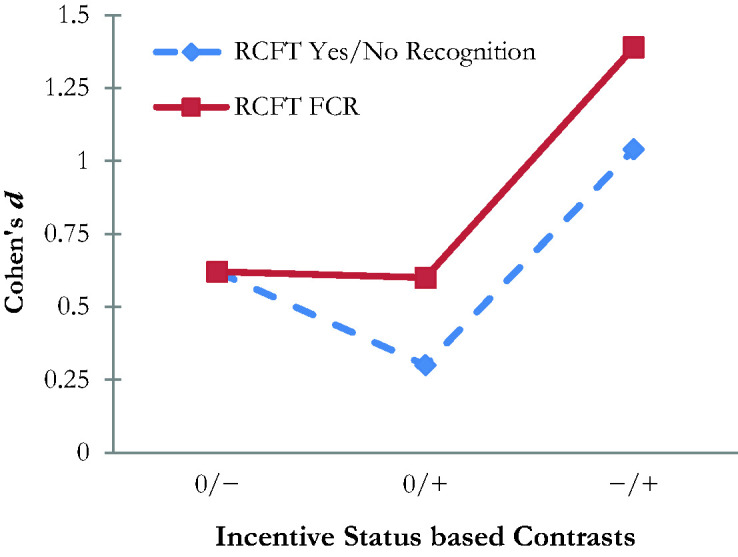
Effect Sizes Associated With Pairwise Contrasts Between Samples as a Function of Incentive Status. 0: Neutral [no incentive to underperform or to perform at maximal ability; represented by the control group (*n* = 80) from the study by Rai et al. (2019)]; −: Negative Incentive [i.e., motivated to underperform; represented by the clinical sample (*n* = 52) from the present study]; +: Positive Incentive [i.e., motivated to perform at maximal ability; represented by the incentivized student sample (*n* = 83) from the present study]; RCFT: Rey Complex Figure Test; FCR: Forced Choice Recognition.

To dissociate the relative contributions of external incentive status and genuine cognitive deficits of the two RCFT recognition scores, we compared our two samples to Rai et al.’s (2019) control group. Our clinical sample had a significantly lower performance on both RCFT recognition trials (*d* = 0.62, medium effect). Our student sample produced a higher mean on both the Yes/No Recognition (*d* = 0.30, small effect) and the RCFT*_FCR_* trial (*d* = 0.62, medium effect) than Rai et al.’s (2019) control group. Our clinical sample had a significantly lower mean on the Yes/No Recognition trial compared to our student sample: *t*(130) = 5.68, *p* < .001, *d* = 1.04 (large effect), and we observed similar results on the RCFT*_FCR_*: *t*(130) = 6.25, *p* < .001, *d* = 1.39 (large effect). Figure 1 provides a visual display of the RCFT*_FCR_*’s differential sensitivity to incentive status.

Results diverged on free-standing PVTs. BR*_Fail_* on the TOMM-1 was significantly higher (40.4%) in our clinical sample compared to our student sample (9.0%): *z*(133) = 4.06, *p* < .001. However, on the WCT the two groups were not statistically different on BR*_Fail_* (29.3–30.8%): *z*(125) = 0.17, *p* = .862.

### RCFT Recognition Trials as Predictors of Cognitive Ability

A visual inspection of the distribution of raw scores for the two RCFT recognition trials across our two samples revealed several important features of these scales. The RCFT*_FCR_* was better at discriminating between the two groups (i.e., students and patients) than was the Yes/No Recognition trial. However, within each sample, the two RCFT recognition trials produced similar cumulative frequency curves.

Correlation coefficients were computed between the Yes/No Recognition trial, the RCFT*_FCR_* and select measures of cognitive ability. Both RCFT recognition trials positively correlated (.45–.55, *p* < .01) with the VPA-3. However, the Yes/No Recognition trial was unrelated to performance on the CDT, while the RCFT*_FCR_* positively correlated with it [*r*(53) = .33, *p* = .018)]. Both trials were correlated with key scores on the HVLT-R. However, only the RCFT*_FCR_* produced a significant correlation with the HVLT-R*_FCR_* [*r*(53) = .37, *p* = .007). Overall, the RCFT*_FCR_* shared more variance with the VPA-3, CDT and the HVLT-R (*r*_xy_^2^: .11–.35) than did the Yes/No Recognition trial (*r*_xy_^2^: .01–.24).

Finally, the classification accuracy of the Copy trial, the Yes/No Recognition and RCFT*_FCR_* trials were computed using the VPA-3, Clock Drawing Test and the Delayed Recall (DR) trial of the HVLT-R as criterion measures (see Table 5). The score on the Copy trial only produced a significant AUC against the VPA-3 (.70; 95% CI: 62–89). It also produced the most stable overall correct classification (.64–.69). The Yes/No Recognition trial was a significant predictor of the VPA-3 and the HLVT-R DR, although overall correct classification was lower (.52–.65). The RCFT*_FCR_* produced the highest AUC (.73–.87; 95% CI: .57–.97) and overall correct classification (.56–.84).

**Table 5.** RCFT Variables as Predictors of Visuospatial Skills and Verbal Memory in the Clinical Sample.

				Criterion variable									
				VPA-3			CDT			HVLT-DR		
				≥5			≥8			≥8		
RCFT				40%			80%			40%		
Variable		Cutoff	BR	SENS	SPEC	ACC	SENS	SPEC	ACC	SENS	SPEC	ACC
Copy	AUC (95% CI)			.70 (.62–.89)			.71 (.46–.96)			.60 (.45–.76)		
		≥28	63%	.47	.80	.64	.70	.73	.72	.39	.67	.53
		≥30	40%	.73	.60	.67	.70	.44	.57	.68	.52	.60
		≥32	31%	.83	.50	.67	.70	.32	.51	.74	.38	.54
		≥34	17%	.97	.40	.69	.80	.17	.49	.87	.24	.56
												
Y/N Rec	AUC (95% CI)			.69 (.52–.82)			.65 (.43–.87)			.72 (.58–.86)		
		≥19	69%	.53	.65	.59	.70	.59	.65	.58	.71	.65
		≥20	54%	.77	.40	.59	.80	.32	.56	.77	.38	.58
		≥21	29%	.83	.20	.52	.80	.17	.49	.87	.24	.56
		≥22	17%	1.00	.10	.55	.90	.02	.46	1.00	.10	.55
FCR	AUC (95% CI)			.82 (.71–.94)			.73 (.57–.90)			.87(.77–.97)		
		≥19	67%	.63	.75	.69	.80	.59	.70	.77	.90	.84
		≥20	50%	.83	.65	.74	.90	.41	.66	.87	.67	.77
		≥21	35%	.90	.50	.70	.90	.29	.60	.87	.43	.65
		≥22	25%	.97	.20	.59	1.00	.12	.56	.97	.19	.58

*Note*. RCFT: Rey Complex Figure Test; Y/N Rec: Yes/No Recognition raw score; FCR: Forced Choice Recognition raw score; VPA-3: Visual-Perceptual Ability Composite (cutoff; CDT: Clock Drawing Test (Rouleau et al., 1992); HVLT-R: Hopkins Verbal Learning Test – Revised (Brandt & Benedict, 2001); DR: Delayed recall raw score; BR: Base rate (%); SENS: Sensitivity; SPEC: Specificity; ACC: Overall accuracy (average of sensitivity and specificity).

### Clinical Implications

Since a score ≤17 on the recognition trials was specific to invalid performance and therefore, an unreliable measure *of cognitive ability*, the Yes/No Recognition and RCFT*_FCR_* scores were effectively seven-point scales (18-24). Attempts to provide demographically stratified standard scores that span across the full spectrum of cognitive functioning (from Impaired to Very Superior) within such a restricted range would likely be fraught with scaling artifacts. Therefore, we propose a three-way clinical classification of Inferior (mild deficits), Within Normal Limits (WNL; intact/average range performance) and Superior (above average), with scores ≤14 considered Invalid, and scores of 15–17 considered Questionable. Reducing a measurement scale to a small number of clinically meaningful categories is a long-standing practice in neuropsychology (Guilmette et al., 2020; Lezak et al., 2012). Of course, the ultimate interpretation will depend on the clinical context, weighing medically verified neuropsychiatric conditions as mitigating factors against the number and level of PVT failures. Within the clinical sample, there was a strong linear relationship between RCFT recognition scores and VPA-3 and HVLT-R DR values (see Table 6).

**Table 6.** Cumulative Percentage (%_CUM_) and Recommended Clinical Classification Ranges for RCFT Recognition Trial Scores within the Student and Clinical Samples.

	Yes/No Recognition				RCFT *_FCR_*				
Raw	Student	Clinical			Student	Clinical			
Score	%*_CUM_*	%*_CUM_*	*M*_VPA-3_	*M*_HVLT_	%*_CUM_*	%*_CUM_*	*M*_VPA-3_	*M*_HVLT_	Classification range
≤12	0	0			0	1.9			Invalid
13	0	0			0	5.8			Invalid
14	0	1.9			0	9.6			Invalid
15	0	5.8			0	11.5			Questionable
16	1.2	17.3			1.2	15.4			Questionable
17	1.2	19.2			2.4	21.2			Questionable
18	6.0	30.8	4.0	25.5	6.0	32.7	1.8	18.8	Inferior
19	12.0	46.2	4.4	36.6	7.2	50.0	5.0	31.4	Inferior
20	30.1	71.2	4.1	37.3	16.9	65.4	3.6	36.6	Within normal limits
21	53.0	82.8	5.3	36.3	26.5	75.0	4.8	41.8	Within normal limits
22	71.1	96.2	4.3	33.0	49.4	90.4	5.3	41.0	Within normal limits
23	95.2	98.1	6.0	40.0	78.3	94.2	5.5	46.5	Superior
24	100.0	100.0	7.0	59.0	100.0	100.0	6.3	46.0	Superior

*Note*. Shading represents the change in confidence in correctly classifying a given score as *invalid* (darker means more likely to be invalid) and delineates the range of performance that should not be interpreted clinically; RCFT: Rey Complex Figure Test; FCR: Forced Choice Recognition raw score; *M*_VPA-3_: Mean score on the Visual-Perceptual Ability Composite; *M*_HVLT_: Mean T-score on the Delayed Recall trial of the Hopkins Verbal Learning Test - Revised; **Invalid**: Scores in this range have not been observed in healthy controls, are rare in clinical patients, and when they do occur, they are associated with failure on other performance validity tests; therefore, they should not be interpreted as evidence of impairment; **Questionable**: Scores in this range are rare in both healthy controls and clinical patients, and when they do occur, they are associated with failure on other performance validity tests; however, in examinees with otherwise valid neurocognitive profiles, they may be considered evidence of impaired visuoperceptual and memory; **Inferior**: Scores in this range are rare in healthy controls, but observed in a third of clinical patients; therefore, provide evidence of mild cognitive deficits; **Within Normal Limits**: About half of the healthy controls and clinical patients scored in this range, indicating intact performance; **Superior**: A score in this range indicates above average performance in healthy controls, and top 5-10% performance in clinical patients.

## Discussion

This study was the first attempt to validate the RCFT*_FCR_* trial as an EVI for a clinical sample. We hypothesized that (a) the RCFT*_FCR_*’s classification accuracy would be attenuated by a confluence of genuine deficits and non-credible performance; (b) RCFT*_FCR_* classification accuracy would be similarly sensitive to natural variability in cognitive ability as the RCFT’s Yes/No Recognition trial; and (c) our student control sample, incentivized to perform well, would produce higher scores on the both the RCFT_*FCR*_ and the RCFT Yes/No recognition trials than had Rai et al.’s (2019) control group who were not incentivized to perform well. Our results provided mixed support for these hypotheses.

AUC values for the two RCFT recognition trials against the TOMM-1 were significantly lower (.60–.64) in our clinical sample compared to the original RCFT_*FCR*_ study (.78–.82). However, while the AUC for Yes/No Recognition was significantly lower against the WCT compared to the original sample (.71 versus .82), the RCFT*_FCR_* achieved essentially the same AUC (.83) as it had in the original study. In fact, the ≤18 cutoff for the RCFT_*FCR*_ produced comparable specificity values (.81–.97 versus .88–.89) while maintaining similar levels of sensitivity (.52–.92 versus .58–.72). Thus, contrary to our expectations, the RCFT*_FCR_* maintained the same classification accuracy in our clinical sample as had been reported by Rai et al. (2019) in the experimental malingering paradigm.

In terms of its sensitivity to fluctuations in cognitive ability, the RCFT*_FCR_* outperformed Yes/No Recognition, explaining a larger proportion of variance (11-35% versus 1–24%) in visuospatial and verbal memory skills and producing superior classification accuracy (AUC: .73–.87 versus .65–.72) as a PVT in our clinical sample. The RCFT*_FCR_* distribution was also associated with a stronger, more refined, and clinically meaningful gradient of difficulty when using the VPA-3 and the HVLT-R DR as references (see Table 6).

Our last hypothesis was fully supported in that our incentivized student sample outperformed Rai et al.’s (2019) control group in their original validation study on both RCFT recognition trials (small-medium effect). However, the RCFT*_FCR_* was actually more sensitive to changes in performance associated with incentive status than was the Yes/No Recognition trial (*d*: .60–1.39 versus .30–1.04).

## Incidental Findings

Our data were largely consistent with the domain specificity effect, in that similarity in the cognitive domain (attention, memory, processing speed, verbal reasoning) or sensory modality (auditory, visual, tactile) between predictor and criterion PVTs influenced classification accuracy (Abeare, Sabelli, et al., 2019; Erdodi, 2019; Schroeder et al., 2019). AUC values for the RCFT based EVIs were consistently higher against the EI-5*_MEM_* (the modality-congruent validity composite) than the EI-5*_PSP_* (the modality-incongruent validity composite), suggesting that instrumentation artifacts may exert a subtle but detectable influence on signal detection analyses. Although both the RCFT Yes/No Recognition and the RCFT*_FCR_* trials performed well across a strategically engineered variability in criterion PVTs, increasing confidence in our overall findings, domain specificity as a potential confound may warrant further research.

Interestingly, the effect of the examinee’s incentive status on the outcome of these free-standing PVTs was instrument specific. Namely, BR*_Fail_* on the TOMM-1 was 4.5 times higher among clinical patients with an incentive to appear impaired than among students with an incentive to demonstrate their best ability. In contrast, there was no difference between these two groups for BR*_Fail_* on the WCT. These findings are consistent with previous reports (Abeare, Erdodi, et al., 2020; Erdodi, Hurtubise, et al., 2018), and they challenge the prominence of the examinees’ external incentive status in diagnostic models for malingering (APA, 2013; Erdodi et al., 2018; Slick et al., 1999). While the new RCFT*_FCR_* trial was more sensitive to the effect of incentive status than the RCFT Yes/No Recognition trial (Figure 1), it must be noted that incentive to appear impaired and elevated risk of genuine neuropsychological deficits were conflated in our clinical sample.

 The unexpectedly high BR*_Fail_* on the free-standing PVTs (9.0%-29.2%) in the incentivized control group puts the low BR*_Fail_* on the Yes/No Recognition and RCFT*_FCR_* trials (1.2%) in perspective. Namely, it neutralizes arguments that EVIs inevitably conflate genuine impairment and non-credible responding (Glassmire et al., 2019; Messa et al., 2020) when compared to free-standing PVTs that are, by design, robust to genuine and severe cognitive impairment (Abeare et al., 2019; Erdodi & Rai, 2017; Whitney et al., 2013). If this finding is replicated by future research, it would further enhance the appeal of the RCFT recognition trials as EVIs.

The improved performance on the RCFT*_FCR_* relative to the Yes/No Recognition trial in the student sample may be due to the fact it controls for variability in the subjective threshold of certainty individuals require to endorse a given shape as a target stimulus. Cautious examinees may choose not to circle Yes/No Recognition items that seem familiar (but are not fully confident in their decision) to avoid making an error. In contrast, knowing that one of the drawings within each pair of the RCFT*_FCR_* is definitely a target makes it easier to select the more familiar item.

## Reflections on Control Group Methodologies

Our a priori, rationally based prediction of relative shrinkage in classification accuracy among patients with genuine cognitive deficits and external incentives to appear impaired was not substantiated. This surprising finding underlines the importance of empirically verifying even intuitive and logically appealing assumptions. Our results supported, instead, previously voiced theoretical concerns (Giromini et al., 2019; McWhirter et al., 2019; van Helvoort et al., 2019) and published data (Abeare et al., 2019; An et al., 2019; Hurtubise et al., 2020; Roye et al., 2019) about the epistemological ambiguity around the incentive status of control groups comprised of undergraduate research volunteers. Researchers long assumed that cognitively healthy university students assigned to the control condition would demonstrate their highest ability level by default. Some newer investigators began to question the validity of this unverified assumption, noting that research participants had been rewarded for their time but not for the quality of the data they produced (An et al., 2017; Powell et al., 2004; Roye et al., 2019; Russeler et al., 2008; Tan et al., 2002). Therefore, the magnitude of their incentive to fully comply with the instructions (i.e., appear impaired without being detected) does not match real-world malingerers who might be incentivized by 7-figure personal injury settlements (Dunn et al., 2003; Grant et al., 2020; Jelicic et al., 2011).

Moreover, emerging evidence suggests that the instructions given to examinees have a weak effect on the credibility of their response sets overall (Abeare, Hurtubise, et al., 2020; Niesten et al., 2017). In other words, reminding real-world patients to provide valid data does not assure that outcome. Likewise, past studies showed that a variable proportion of student volunteers who were assigned to the control condition and asked to demonstrate their best ability in academic research settings failed PVTs (An et al., 2012; DeRight & Jorgensen, 2015; Ross et al., 2016; Roye et al., 2019; Santos et al., 2014; Silk-Eglit et al., 2014). Ironically, participants assigned to the experimental malingering condition also occasionally demonstrated intact cognitive ability – in other words, they *failed at failing* (Abeare et al., 2020).

Essentially, past performance validity research studies using the experimental malingering paradigm specifically and relying on student volunteer participants generally, were subject to several internal and external validity threats. There seems to be an emerging consensus that there is no guarantee that any given research participants will comply with study instructions. An et al. (2017) went as far as to suggest that an incentive to appear impaired and a lack of incentive to perform well are similar motivational states. Similarly, criterion grouping in studies based on experimental malingering can be considered a pseudo-independent variable (Hurtubise et al., 2020), as the only control investigators have in this circumstance is through instructions given, but does not extend to whether those instructions are executed.

Results from the current study further addressed the credibility of psychometric data produced by cognitively healthy university students. Despite (a) salient demand characteristics (i.e., administering tests in a classroom setting and repeatedly emphasizing the educational value of full engagement) and (b) a performance-based reinforcement contingency (i.e., full points only awarded for valid responses), a surprisingly high proportion of our student controls (29.3%) failed the WCT, a free-standing PVT. This failure rate is twice as high as the rate of non-credible profiles in clinical and even forensic settings (Young, 2015), and it far exceeds findings by previous research on performance validity among undergraduate research volunteers (An et al., 2017; DeRight & Jorgensen, 2015; Ross et al., 2016; Santos et al., 2014; Silk-Eglit et al., 2014). In the context of a 9% failure rate on the TOMM-1 and a 1.2–2.4% failure rate on the RCFT recognition trials, these high WCT failure rates seem to be an isolated anomaly that serve as an important reminder that external incentives fail to explain a significant amount of variance in PVT failures. In fact, since normative data for well-respected tests have not been screened for non-credible responding, instances of invalid performance can even shift normative data toward impairment and inflate error variance in clinical decision making (Erdodi, Hurtubise, et al., 2018).

Despite these general concerns, we found a comparable medium effect size for RCFT recognition trials when comparing classification accuracies of our incentivized control group and controls from the original study (Rai et al., 2019). However, the effect size for the contrast between controls and clinical patients doubled when the comparison was based on students who were motivated to do well. Such discrepancies may have important implications for high-stake research studies (randomized clinical trials, pharmacological research). More importantly, they suggest that, however imperfect, calculated efforts to “sanitize the sample” (i.e., reduce experimental confounds) can improve data quality. Additionally, our results should sensitize research consumers to the issue of performance validity even among controls who have no apparent reason to underperform.

## Clinical Applications

Our results support the use of the RCFT*_FCR_* as an EVI for clinically referred patients, especially since the RCFT*_FCR_* showed potential to double as a valid measure of actual visuospatial memory. The RCFT*_FCR_* demonstrated superior overall psychometric properties and provided unique information about the credibility of the response set, complementing the established use of the Yes/No Recognition trial as an EVI (Blaskewitz et al., 2009; Lu et al., 2003; Sugarman et al., 2016). The RCFT*_FCR_*’s low cost (open source, quick and easy to administer and score) and its potential dual-purpose make it a valuable addition to a standard neuropsychological test battery. The distribution of RCFT recognition scores revealed an important scaling artifact. Essentially, when any score ≤17 was considered invalid, clinicians were left with just a seven-point performance range (18-24) that was free of the “invalid before impaired paradox” (i.e., a score being deemed *invalid* before a credible deficit can be interpreted; Erdodi & Lichtenstein, 2017). Because such a restricted range does not likely allow for meaningful, demographically adjusted T-scores, we propose that clinicians adopt an interpretive trichotomy (Inferior – WNL – Superior), following differential base rates between an incentivized control group and a clinical sample (Table 6). Fortunately, because performance on RCFT recognition trials was unrelated to age, sex and level of education, raw scores in these analyses seem empirically justified. Naturally, this restricted 7-point range constrains the clinical utility of the RCFT*_FCR_* as a measure of visual recognition memory. It also makes the distributional properties and therefore, the clinical interpretation of given scores vulnerable to fluctuations across samples. Although the RCFT*_FCR_* may be unfit as a fine-tuned measure of memory functioning, it retains incremental validity over the Yes/No Recognition trial – both as an EVI and as an ability test. In fact, many empirically validated and widely used cognitive screening tests have similarly steep item-characteristic curves with psychometrically inactive left tails (Erdodi, Shahein, et al., 2020; Hilsabeck et al., 2015; Hoops et al., 2009). Of course, replication in larger samples is needed to determine whether these findings generalize to other populations.

## Strengths, Limitations, and Directions for Future Research

By extending our investigation to clinical patients and adding an incentivized control group, we addressed several limitations of prior studies that were based on an experimental malingering paradigm (Abeare, Hurtubise, et al., 2020; Erdal, 2004; Niesten et al., 2017). We incorporated two of the same criterion PVTs from the original study (Rai et al., 2019) into the present analyses, permitting a direct comparison between past and present research. In addition, we tested two new validity composites with an engineered method variance to rigorously cross-examine RCFT recognition trials and protect against instrumentation artifacts (Erdodi, Hurtubise, et al., 2018; Erdodi, Tyson, et al., 2018).

The most salient limitation of the study was its small sample size and the composition of the clinical sample. Our predominantly white patients from a single geographic region, referred for disability evaluations may represent a unique set of sample characteristics that limit the generalizability of these findings to other populations (Kura, 2013; Leon & Leon, 2014; Lichtenstein, et al., 2019; Lynn, 2010) with different medical etiologies and incentive structures (Chafetz, 2011; Fuermaier et al., 2019; Giromini et al., 2019; Harrison, 2017; Merten & Rogers, 2017; van Helvoort et al., 2019) and different demographics (race, level of education, socio-economic status). Also, our group administration format for the RCFT within the student sample may have altered the psychometric properties of the instruments. Finally, the validation of the RCFT*_FCR_* trial as a measure of visual-perceptual memory was incomplete, as we merely provided a proof of concept for the RCFT_*FCR*_ as an ability measure. Indeed, the absence of an independent, well-established test of visual memory with a separate recognition trial as a criterion measure is a notable limitation that should be addressed in future research. Before the instrument can be recommended for clinical use, it requires replication using larger, clinically and demographically more diverse samples against established measures of visual memory.

## Conclusion

Our results suggest that previously published validity cutoffs on the RCFT*_FCR_* trial maintain high specificity to psychometrically defined non-credible responding among clinical patients, and were not fully redundant with decisions made from cutoffs on the Yes/No Recognition trial. As such, the RCFT*_FCR_* provides unique and relevant information for performance validity assessment. Moreover, the RCFT*_FCR_* had a stronger correlation with visual-perceptual and verbal memory skills than the Yes/No Recognition trial. Even if limited to interpreting valid clinical data from a seven-point scale (i.e., scores of 18–24), both RCFT recognition trials may provide useful clinical information about cognitive functioning. Future research would benefit from further exploring the RCFT*_FCR_*’s clinical utility and in its use among children (Lichtenstein et al., 2017, 2018, 2019). Replication using geographically and demographically diverse samples with a wide range of medically verified neuropsychiatric conditions is needed to determine the generalizability of the current findings.
